# Deep Sequencing Analysis Identified a Specific Subset of Mutations Distinctive of Biphasic Malignant Pleural Mesothelioma

**DOI:** 10.3390/cancers12092454

**Published:** 2020-08-29

**Authors:** Federica Torricelli, Filippo Lococo, Teresa Severina Di Stefano, Eugenia Lorenzini, Simonetta Piana, Riccardo Valli, Ottavio Rena, Giulia Veronesi, Andrea Billè, Alessia Ciarrocchi

**Affiliations:** 1 Laboratory of Translational Research, Azienda Unità Sanitaria Locale-IRCCS di Reggio Emilia, 42123 Reggio Emilia, Italy; federica.torricelli@ausl.re.it (F.T.); eugenia.lorenzini@ausl.re.it (E.L.); 2 Thoracic Surgery, Fondazione Policlinico Universitario A. Gemelli, IRCCS, Università Cattolica del Sacro Cuore, 00168 Rome, Italy; filippo_lococo@yahoo.it; 3 Unit of Thoracic Surgery, Azienda Unità Sanitaria Locale-IRCCS di Reggio Emilia, 42123 Reggio Emilia, Italy; teresadistefano91@gmail.com; 4 Pathology Unit, Azienda Unità Sanitaria Locale-IRCCS di Reggio Emilia, 42123 Reggio Emilia, Italy; simonetta.piana@ausl.re.it (S.P.); Riccardo.valli@ausl.re.it (R.V.); 5 Thoracic Surgery Unit, University of Eastern Piedmont, 28100 Novara, Italy; ottaviorena@libero.it; 6 Unit of Thoracic Surgery, Humanitas Cancer Center, 20132 Milan, Italy; giulia.veronesi@humanitas.it; 7 Division of Cancer Studies, King’s College London, Guy’s Hospital, London SE1 9RT, UK; andrea.bille@gstt.nhs.uk; 8 Department of Thoracic Surgery, Guy’s Hospital, London SE1 9RT, UK

**Keywords:** Malignant Pleural Mesothelioma, histotype, genetic mutations, next generation sequencing

## Abstract

Malignant Pleural Mesothelioma (MPM) is a heterogeneous disease. Morphologically, three different phenotypes are distinguishable: epithelioid (e-), sarcomatoid (s-) and biphasic (biph-) MPM, the latest, being a mixture of e- and s-MPM cells. Being an intermediate entity, management of biph-MPM, remains debatable and controversial, with different guidelines recommending distinct approaches. Identification of biph-MPM associated genetic alterations, through deep sequencing analysis, may provide useful tools to understand these lesions. A retrospective cohort of 69 surgically resected MPMs, 39 biph-MPMs (56.5%) and 30 e-MPMs (43.5%) was selected. A separate set of 16 biph-MPM was used as validation set. Deep sequencing analysis on an MPM-specific custom panel (MPM_geneset) comprising 1041 amplicons spanning 34 genes was performed. A total of 588 variants and 5309 mutational events were detected. In total, 91.3% of MPMs showed at least one mutation and 76.8% showed co-occurrence of more than one alteration. Mutations in MXRA5 (*p* = 0.05) and NOD2 (*p* = 0.018) were significantly associated with biph-MPM both in the training and validation cohort and correlated with the extent of the sarcomatoid component. Mutations in NOD2 and XRCC6 correlated with patients’ survival. We demonstrated that biph-MPM are associated with a specific mutation set, and that genetic analysis at diagnosis may improve patients’ risk stratification.

## 1. Introduction

Malignant Pleural Mesothelioma (MPM) is a rare and highly aggressive form of cancer, originating from the mesothelial cells lining the pleural cavity [1]. MPM development is tightly linked to both primary and secondary asbestos exposure [2,3]. The widespread use of these silicate minerals in the mid-20th century and the long latency of this disease makes MPM a current global health issue. MPM is almost universally lethal, and the median overall survival for these patients is currently estimated around one year [4]. 

Current standard treatments for MPM patients have shown only modest survival improvement in the past decades, highlighting the need of new therapeutic options against this disease [5,6].

Understanding the genetic asset of MPM may provide new clues on the molecular basis of this cancer and identify new targeted therapies and effective options. As for many other cancer types, MPM is not a homogenous disease. Histologically, three main classes of MPM have been recognized: epithelioid (e-MPM), sarcomatoid (s-MPM) and biphasic MPM (biph-MPM) which shows the coexistence of epithelioid and sarcomatoid area [7,8,9]. This classification primarily takes into account the morphology and degree of MPM cells differentiation but largely reflects the clinical aggressiveness with sarcomatoid variants being the most aggressive and lethal form of MPM [10,11].

Furthermore, reflecting clinical aggressiveness, histology is currently one of the most relevant criteria for the choice of treatment [12]. While guidelines are quite defined for epithelioid and sarcomatoid lesions, the management of biph-MPM, remains extremely debatable and controversial, with different guidelines recommending distinct approaches for these lesions that together represent about 30% of MPM. This discrepancy largely lines up on the lack of precise information about biph-MPM in the literature. We recently suggested that a multimodal approach, including chemotherapy and cancer-directed surgery, could be associated with improved long-term results in very selected patients with biph-MPM [13]. However, being a mixture of both epithelioid and sarcomatoid components, biph-MPMs are rarely identified on diagnostic pre-surgical biopsies (high discordance rate), which often show only a limited part of the entire disease, with the consequence that biph-MPM are often misdiagnosed at this stage, further impacting on the subsequent patient treatment choice [10,11]. 

The identification of biph-MPM associated prognostic markers, through the employment of deep sequencing analysis, may provide useful tools to refine risk based-stratification of biph-MPM patients, guiding to a more aware clinical management of these patients. This prognostic stratification could help the physicians to plan the best strategy of care, reserving a more aggressive treatment to biph-MPM-patients with better chance of survival while a palliative care could be preferred to improve quality of life in the others. 

Here, we used a deep sequencing approach to investigate the genetic profiles of a cohort of MPM using a MPM-specific gene panel, with the intent of improving our knowledge of the alterations associated with this cancer and in particular to address the complexity of biph-MPMs and to define the impact of the genetic asset on clinical presentation and prognosis of these tumors.

## 2. Results

### 2.1. Clinical Features

A multicentric, retrospective case-control cohort of 69 surgically resected MPMs, comprising 43.5% e-MPM (*n* = 30) and 56.5% biph-MPM (*n* = 39), was retrieved and included in this study. 

Demographics, clinical and pathological features are summarized in Table S1. Asbestos exposure was available for 52 patients. Of these 46 (88.5%) experienced either direct (*n* = 40, 77%) or indirect (*n* = 6, 11.5%) exposure to asbestos. Twenty-five patients (41.7%) were smokers and 9 patients (13%) underwent neoadjuvant treatment. At the moment of diagnosis, approximately half of them presented with an early-stage T1-T2 tumor (56.5%) and 2.9% displayed distant metastasis. In the present cohort, about 60% of patients underwent surgical biopsy only through video-assisted thoracoscopy (or mini-thoracotomy) while in the remaining cases, a cancer-directed surgery (pleurectomy/decortication) was performed (in 9 cases following an induction chemotherapy) with a curative intent. 

Median follow-up for this cohort was 13 (range 0–74) months. The 1-year, and 3-year overall survival rates in the study population were 51% and 13%, respectively.

### 2.2. Genetic Features 

We performed a deep sequencing analysis on a custom panel of 1041 amplicons covering a total of 90.875 bp, spanning 34 genes selected as frequently mutated in MPM based on an accurate revision of the literature (MPM_geneset) [14]. We detected 588 variants and 5309 mutational events within the MPM cohort. Restricting the analysis to non-synonymous, non-intronic mutations with frequency in ExAC in the general population <0.1%, 205 variants and 260 mutational events were selected (Table 1). Normalizing the number of mutations on the length of the analyzed regions covered by the panel we estimated 40.5 mutations for megabase. 

**Figure 1:**
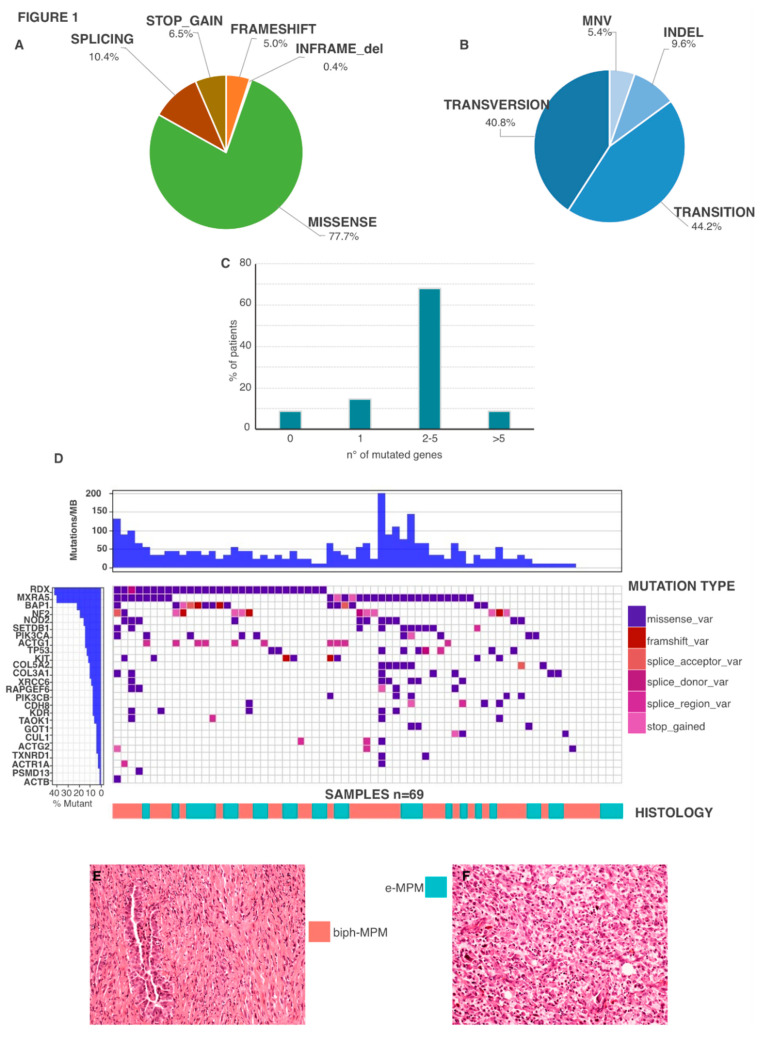
Mutational Profile of the 69 MPMs in analysis. (**A**) Distribution of the somatic mutation events detected in MPMs according with nucleotide substitution. (**B**) Distribution of somatic mutation events detected in MPMs according with predicted functional effects. (**C**) Frequency distribution of the number of mutated genes per patient. (**D**) Waterfall plot representing the gene mutations occurred in each patient (columns). Squares of different colors indicate different mutation types. Upper histograms represent the estimated number of mutations per Megabase for each patient (**E**,**F**). Colored lines in the bottom discriminates patients with different histotypes.

Of these, the vast majority (77.7%) led to amino acid substitution, while stop-gained, frameshift, splice variants and inframe deletion accounted for smaller percentages (6.5%, 5%, 10.4%, and 0.4% respectively), (Figure 1A). Gene amplification could not be evaluated using this approach. Detected mutations were transitions (44.2%), followed by transversions (40.8%%), indels (5.4%) and multi-nucleotide variants (5.4%) (Figure 1B). Figure 1C shows the distribution of the number of mutated genes for patient. 63 MPM (91.3%) showed at least one mutation and 76.8% showed the co-occurrence of more than one mutation (up to 13 different mutations), whereas only 6 patients (8.7%) showed no alterations in the analyzed genes. Figure 1D–F reports the occurrence of gene mutations per each sample ranked by frequency on the overall cohort. Surprisingly, RDX has the highest prevalence of protein-altering mutations in our cohort (29/69, 42%) followed by MXRA5 which was mutated in 40.6% of analyzed samples (*n* = 28). We found 42 mutations for RDX and 47 for MXRA5. Of these the vast majority were missense variants (97.2% RDX, 93.3% MXRA5). One splicing related mutation was observed for RDX while two stop gain mutations were detected in MXRA5. BAP1 and NF2 mutations were detected in 21.7% (*n* = 15) and 18.8% (*n* = 13) of samples respectively, in line with previous reports that describe these as the most frequent mutated genes in MPM [15,16]. Mutations in TP53, CUL1, PIK3CA and TAOK, which were previously reported to be variably mutated in MPM, were observed with frequency in line with previous reports [14,15,16]. No significant correlations were observed between single mutated genes or co-occurring mutations and MPM stage (Table S2, Figure S1).

### 2.3. Genetic Mutations in Biph-MPM

**Figure 2:**
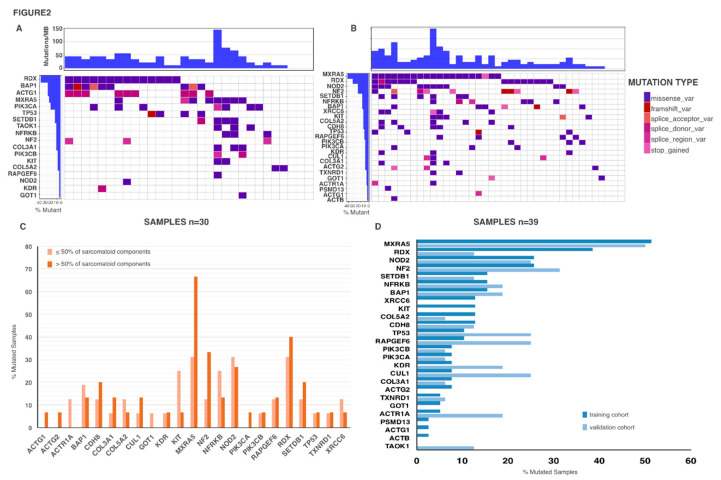
Biph-MPMs have a distinct mutational profile as compared with eMPM (**A**) Waterfall plot representing the gene mutations occurred in each patient with epithelioid MPM (columns). (**B**) Waterfall plot representing the gene mutations occurred in each patient with biphasic MPM (columns). Squares of different colors indicate different mutation types. Upper histograms represent the estimated number of mutations per Megabase for each patient. (**C**) Comparison of mutated genes frequencies between biphasic MPMs with different percentage of sarcomatoid component. (**D**) Comparison of frequencies of the principal mutated genes in biphasic MPM between training and validation cohort.

Next, we compared the genetic profile of biph-MPM with the one associated with e-MPM with the intent of defining the existence of a biph-MPM genetic signature (Table 2). Cohort separation into histotypes showed a remarkable difference in mutation occurrence between biph-MPMs and e-MPM (Figure 2A,B). While mutations in RDX are quite homogeneously distributed in the two subsets, mutations in MXRA5 and NOD2 are significantly associated with biph-MPM (*p* = 0.050 and *p* = 0.018 respectively). MXRA5 is mutated in 51.3% (20/39) of biph-MPM vs. 26.7% (8/30) of e-MPM. Mutations in NOD2 are observed in 25.6% (10/39) of biph-MPM vs. 3.3% (1/30) of e-MPM. Even if not significant, mutations in CDH8 and XRCC6 showed a positive trend of association with the biph-MPM subset. By contrast, mutations in ACTG1 (*p* = 0.002), TAOK1 (*p* = 0.077) and PIK3CA (*p* = 0.090) were more frequent in the e-MPM samples as compared with the other subtype. To complete this set of data we explored the occurrence of these mutations in a retrospective cohort of 11 sMPMs (Tables S3 and S4). We observed that sMPMs share at least in part biph-MPMs genetic alterations. In particular, mutations in MRXA5 and NOD2 were equally occur in sMPM as in biph-MPM even if with slightly different frequencies. By contrast mutations in CDH8 and XRCC6 were detected exclusively in the biph-MPM samples (Table S5). For definition, Biph-MPMs display mixed e-MPM and s-MPM components even if in different extent. We explored the potential correlation of detected mutations with the percentage of s-MPM within each biph-MPM sample (Figure 2C). Mutations in MXRA5 were significantly associated with an increased s-MPM component, indicating that these mutations may be part of the progression process that lead to transdifferentiation of epithelioid MPM cells into the sarcomatoid phenotype. To validate our observations, we analyzed the genetic profile of a separate set of 16 biph-MPM (Figure S2). Noticeably, we observed a quite overlapping distribution of the mutations between training and validation set, strongly confirming the validity of our analysis. In the validation set, MXRA5 and NOD2 scored as the most frequently mutated genes in biph-MPMs (Figure 2D). 

### 2.4. Prognostic Value of Biph-MPM-Associated Genetic Mutations

Univariate analysis performed on biph-MPM showed the prognostic impact of clinical and pathological variables. In detail, the performance status (HR: 3.89, 95% C.I.:1.44–10.49, *p* = 0.007) and the performing of a cancer-directed surgery (HR: 0.43, 95% C.I.: 0.20–0.95, *p* = 0.036) significantly influenced long-term survivals (Table 3) in accordance with previous data [13]. Next, we aimed to define whether somatic mutations frequently associated with biph-MPM may also have a prognostic value in anticipating clinical aggressiveness. To this end, we investigated the potential correlation of mutations in MXRA5, NOD2, CDH8 and XRCC6 (that display the highest association with this histotype) with survival within the set of 39 biph-MPM included in our cohort. During follow up (mean time 13.6 months, range 1–49), 34 patients (87.2%) died because of disease. Of these, 20 patients showed mutations in at least one of the selected genes. Univariate analysis showed that XRCC6 was significantly associated with survival in biph-MPM while no significant association was identified for MXRA5 and CDH8 and NOD2. This association remained also in a multivariate analysis considering the mutational status of these four genes. 

**Figure 3:**
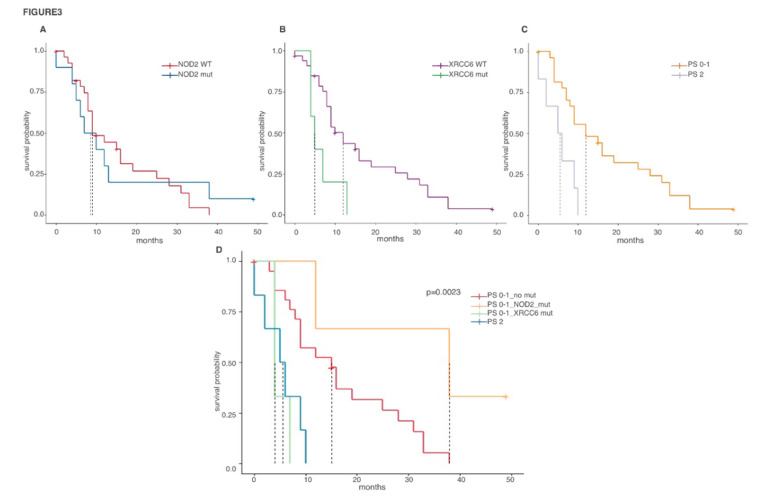
Genetic alterations affect clinical behaviour of biph-MPM independently from performance status. (**A**) Kaplan Meier curves of biph-MPM patients according to NOD2 mutational status. (**B**) Kaplan Meier curves of biph-MPM patients according to XRCC6 mutational status. (**C**) Kaplan Meier curves of biphasic MPM patients according to performance status score. (**D**) Kaplan Meier curves of biphasic MPM patients with different performance and mutational status.

We recently showed that TNM stage, surgery, forced expiratory volume in 1 second (FEV1) and performance status (PS) are clinical variables with a significant impact on biph-MPM patients’ survival. Thus, we performed a multivariate analysis to investigate the weight of both genetic alterations (in the four genes) and clinical features on biph-MPM outcome. Mutations in XRCC6 and high value of performance status significantly correlated with reduced survival in both univariate and multivariate analysis. By contrast, mutations in NOD2 do not correlate with survival probability in the univariate analysis but, when considered in a multivariable model, show a significant protective effect on patients’ survival (Table 3). In fact, Kaplan-Meier plots showed that in patients with a favorable performance status, mutations in NOD2 are associated to an increased survival while mutations in XRCC6 had a significant negative effect on the outcome of these patients which was comparable with the effect determined by an unfavorable PS. (Figure 3).

## 3. Discussion

MPM is a highly deadly disease for which molecular tools are urgently required to improve both patients’ stratification and therapeutic strategies [5,6].

MPM is a heterogeneous entity in terms of morphology, transcription profile and genetic landscape. Asbestos which is the main risk factor for MPM significantly contributes to this heterogeneity, being causative of a wide range of molecular aberrations [3]. In spite of the fact that a significant number of studies investigated genetic and molecular profiles of MPM, definitive and unique markers for this disease are still to be identified. 

Morphological heterogeneity in MPM is evidenced by the existence of two distinct phenotypes (e-MPM and s-MPM) which represent the two extremes of a transdifferentiation process that converts pleura epithelioid cells into mesenchymal-like sarcomatoid cells [7,8,9]. Indeed, recent gene expression studies, identified Epithelial Mesenchymal Transition (EMT) as one of the main drivers of s-MPM evolution and EMT associated proteins as selective marker of either e- or s-MPM [17,18,19]. 

Furthermore, this classification tightly reflects clinical behavior having the two MPM subtypes different progression time and prognosis [10,11]. Still, such dichotomic classification appears to be limited and insufficient to correctly stratify the entire spectrum of morphological heterogeneity of MPM leaving biph-MPM without an appropriate management strategy. Lying in between, prognosis and management of biph-MPM are generally complicated and the lack of specific guidelines leaves the therapeutic strategies of these tumors to the choice of single centers and clinicians. Furthermore, being a mixture of epithelioid and sarcomatoid cells, the diagnosis of biph-MPM is complicated and can be corrupted by the limited representativeness of bioptic samples. This issue has been recently addressed by Blum and colleagues who used a multi-omics approach to quantify MPM heterogeneity [20,21]. Using deep transcriptomic profiling, they showed that e-MPM and s-MPM clusterized as separate entities at the extreme side of the gene expression spectrum, while biph-MPMs do not form a separate meta-cluster but spread in the continuum between e- and s-MPM, suggesting that these lesions are just mixed shadows of the two extreme phenotypes. Based on these results, the Authors propose to revise MPM pathology in terms of molecular gradients and to consider each biph-MPM as a mixture of these two components in which the representativeness of each component affect positioning into the linear spectrum that goes from e-MPM to s-MPM. Establishing thresholds of risk along the gradient will help defining the most appropriate management strategy for biph-MPM patients. However, while fascinating, the possibility of using a “continuum” classification in the daily clinical practice appears rather problematic both in terms of feasibility and interpretation. In this framework, improving the knowledge about the molecular and genetic asset of these tumors is a matter of fundamental importance for physicians involved in MPM care.

Therefore, in the present work, we attempted to address this point by exploring genetic alterations in a retrospective cohort of MPM comprising a large set of biph-MPMs. We showed that while sharing common MPM genetic alterations (like BAP1 and NF2) with e-MPM, biph-MPM are characterized by a peculiar set of mutations. Both in the training and validation set, mutations in MXRA5 and NOD2 are significantly associated with the biph-MPM while alterations in XRCC6 and CDH8 seem to be enriched in this subset of MPM. Noticeably, these genes have all been associated with cancer development and progression [15,22,23,24,25]. 

Mutations in MXRA5 were previously described in MPM. This gene code for a protein involved in extracellular matrix remodeling and as such likely in cell shape reorganization movement, migration like the one pleural cells undergo in the transition from the e- to the s-phenotype. Indeed, MXRA5 has been shown to be a target of TGF involved in the regulation of inflammation and fibrosis in chronical diseases [26] and it has been suggested as prognostic biomarker in colorectal and non-small cell lung cancer [27,28]. Besides MPM, mutations in MXRA5 were previously reported in liposarcoma, colon and lung cancer. The fact that mutations in MXRA5 may be associated with transition from e- to s- phenotype is further confirmed by the positive association we observed with the s-component percentage in the tumor. 

The NOD2 gene code for a transmembrane receptor of the Nod1/Apaf-1 family and encodes a protein with two caspase recruitment (CARD) domains and six leucine-rich repeats (LRRs) [29]. NOD2 is involved in the regulation of innate immunity, and once activated triggers induction of the MAPK and NFKB pathway [30,31]. Its function within the immune-system is intriguing since, our survival analysis indicated that mutations in NOD2 have a protective role in MPM being associated with improved survival probability independently of the PS. Thus, even if further studies are needed, we may speculate that NOD2 has an inhibitory role immune-response against MPM that may be partially overcome by inactivating mutations. 

CDH8 and XRCC6 are engaged in cell movement ad DNA repair respectively [32,33]. Our data seem to indicate that mutations in XRCC6 may be relevant in dictating biph-MPM prognosis. Taking into consideration the role of XRCC6 and its relevance in maintaining DNA homeostasis, it is not surprising that mutations in this gene may be associated with increased DNA damage and therefore increased aggressiveness, as it has already been shown in other cancers. 

Genetic markers of aggressiveness and survival in MPMs are hardly been described. Indeed, several studies exploring the genetic landscape of MPMs indicated that clinical behavior of MPM is only marginally influenced by genetic alterations as, differently from other contexts, a very limited number of recurrent mutations are described in this type of tumor [14,15,16]. By contrast, epigenetic events seem to have a stronger impact on clinical behavior of MPM as compared to genetic mutations [34,35]. Similarly, our data, while showing trends of association, does not identify relevant alterations that alone may be relevant in predicting patients’ outcome. In addition, the reduced size of the cohort and the heterogeneity in the protocol of treatments of patients enrolled in this study further complicate this type of evaluation. 

On one hand, our work has the merit of having focused specifically on the biph-MPM subtype providing new information about the genetic asset of these tumors. On the other hand, we are aware of the limitations of our study among which is the use of a target gene panel strategy for the genetic profiling. We acknowledge that the use of a wider approach like whole exome sequencing (WES) would allow a deeper evaluation of the genetic asset of our study set. However, as the results of previous WES study in MPM demonstrated, MPMs are characterized by a low mutational burden and low genetic complexity, with very few significantly recurrent mutations. In this regard, our panel, designed on the basis of careful literature revision, has been developed to cover the most frequent alterations in MPM, thus ensuring a significant coverage of the genetic events within our cohort. 

This is a retrospective multicentric study which focus on a limited cohort of MPMs and, our results could be affected by potential selection biases as well as statistical limitations due to sample size. Still in spite of this, our data seem to support the idea that biph-MPMs are characterized by a “peculiar” set of genetic alterations and that genetic assets may affect (at least in part) the biph-MPM behavior. In particular, the genetic mutations we found associated with biph-MPMs may become precious diagnostic tools helping to discriminate biph-MPM from e-MPM in pre-surgical biopsies, overcoming the potential limitation of the representativeness of the sampling always associated with this type of procedure. Moreover, from a theoretical point of view, the creation of a gene panel able to predict prognosis in biph-MPM patients, could be of great help for the physicians in planning the best strategy of care. In fact, more aggressive treatments could be performed in a cohort of biph-MPM with a favorable genetic asset while in the remaining cases with a dismal prognosis the strategy of care could be more focused on the palliation of symptoms and on the preservation of an acceptable quality of life.

## 4. Materials and Methods

### 4.1. Patients Selection

A retrospective multicentric cohort of MPM, enriched in biph-MPM samples, were retrieved from the participating institutions. Inclusion criteria were: Age at diagnosis >18, histological diagnosis of e-MPM or biph-MPM, formalin fixed paraffin embedded (FFPE) material available for the analysis. Exclusion criteria were: histological or cytological diagnosis of sarcomatoid MPM, uncertain diagnosis of non-sarcomatoid MPM, missing data on stage or treatment. Revision of the histological slides was performed by a designated expert thoracic pathologist to confirm the final diagnosis. According to the WHO recommendation, tumors containing at least 10% of each component (epithelioid or sarcomatoid) are classified as having biphasic histology [36]. The 8th TNM classification system was adopted and the surgical-pathological stages (re)assigned accordingly [37]. Detailed diagnostic and therapeutic strategy information regarding this cohort were reported in our previous manuscript [13]. The study protocol was approved by the Institutional Review Board of the Azienda Unità Sanitaria Locale—IRCCS di Reggio Emilia, Reggio Emilia, Italy and by the Local Ethical Committee AVEN (protocol number 2017/0013216, 23 May 2017. Project identification code: Biph-MPM) 

All the subjects included in the study provided their informed consent to participate. The study was conducted in accordance with the Declaration of Helsinki.

### 4.2. Next Generation Sequencing

DNA from bioptic or surgical FFPE specimens was extracted using Maxwell nucleic acid extractor (Promega) and quantified using Qubit dsDNA HS assay kit (ThermoFisher) as previously described [38,39]. Truseq custom amplicon low input kit (Illumina) was used for library preparation. The genes included in the panel were summarized in Table S6. Sequencing was performed on Illumina Nextseq 500 Mid output reagent cartridge v2 300 cycles (2 × 151). Primary bioinformatic analysis was performed on Illumina Basespace environment using Amplicon DS software. Variant studio software (Illumina) was used to visualize .vcf files, investigate mutations occurred in each sample, annotate them and apply selection filters. Mutations were considered reliable if the locus presented a minimum coverage of 1000×.

### 4.3. Statistical Analysis

All analysis performed in this study were elaborated using R software. Waterfall plots were generated using R “GenVisR” package. Analysis of association between genes mutational status and clinical features were performed applying Fisher test for categorical variables and Kruskal Wallis test for continuous variables.

Classification tree was generated using Orange Canvas software. Mutational status of genes altered in at least 10% of patients were included as attributes and the binary tumor stage (TNM Stage I vs. TNM Stage II–IV) defined the two decision classes. The AUC (Area under the curve) of the model was calculated after 10-fold cross validation.

Survival analysis was performed applying Cox proportional hazard model and Kaplan Meier curves were generated using R “Survminer” package. Associations and differences were considered statistically significant with a *p* value < 0.05.

## 5. Conclusions

In conclusion, we identified a panel of 4 genes preferentially and frequently altered in biph-MPM, which may represent a useful tool for a correct diagnosis of these bi-valent tumors. Furthermore, we showed that genetic analysis at the time of diagnosis may improve risk-based stratification of patients adding information to the evaluation of clinical features that, like performance status, condition patient outcome.

## Figures and Tables

**Table 1:** List of mutations detected in epithelioid and biphasic MPM patients.

Gene	Mutations	Total Number of Mutation	Number of Mutated Patients
ACTB	p.Val45Leu	1	1
ACTG1	c.984+7_984+9delCGA	10	10
ACTG2	p.Pro113Ser; p.Gln354Ter; c.255+7G>A	3	3
ACTR1A	p.Val47Ile; c.751-7C>T	2	2
BAP1	p.Cys39PhefsTer33; p.Gln277Ter; p.Tyr241Ter; p.Asp68Gly; p.Leu180Arg; p.His169Arg; p.Asp74Tyr; c.68-1G>A; p.Glu154Asp; p.Asn229Lys; p.Arg300GlyfsTer6; p.Phe170Val; p.His141Tyr; p.His169Gln; p.His141Arg; c.581-2A>G; p.His141Pro	17	15
CDH8	p.Pro616Gln; p.Pro214His; p.Asn609Lys; p.Ala174Thr; p.Val636Phe	5	5
COL3A1	p.Pro49Thr; p.Asn1282Lys; p.Pro135His; p.Pro904His; p.Arg1024Gln; p.Pro89Leu; p.Gly633Val	7	6
COL5A2	p.Gly489Cys; p.Gly804Asp; p.Pro118Thr; p.Gly570Asp; p.Gly1212Asp; p.Gly819Ser; p.Leu198Phe	7	7
CUL1	p.Leu92Ile; c.316-8G>T; c.2251-7C>T	3	3
GOT1	p.Asp64Glu; p.Gln105Ter; p.Arg160His	3	3
KDR	p.Arg1118Gln; p.Asp623Tyr; p.Glu540Ter; p.Ser427Tyr; p.Ser1260Asn; p.Leu383Met; c.162-3C>T	7	4
KIT	p.Pro41Thr; p.Glu198Lys; c.926-1G>T; p.Gln190His; p.Phe522Leu; p.Gly297Glu; p.Leu305Phe	7	7
MXRA5	p.His2434Gln; p.Leu1313Phe; p.Thr468Ile; p.Thr1507Ile; p.Leu1413Ile; p.Arg1232Ile; p.Pro1112Thr; p.Arg541Trp; p.Glu1540Lys; p.Ala1601Thr; p.Ala1541Thr; p.Arg791Cys; p.Pro1171Ser; p.Asp734Asn; p.Ala557Val; p.IleSerProPro824ValSerProArg; p.Glu230Ter; p.Thr2823Ile; p.Ala623Thr; p.Ala1567Thr; p.Gly733Arg; p.His268Asn; p.Gly1597Glu; p.Pro1516Leu; p.Leu2284Met; p.Trp2277Cys; p.Pro1788Leu; p.Ser1199Phe; p.Gly160Val; p.His2498Asn; p.Ala1187Thr; p.Pro1289Ser; p.Ser1147Phe; p.Ala829Val; p.Ala1512Thr; p.Cys275Phe; p.Asp2230Asn; p.Ser1811Phe; p.Pro1259Leu; p.Ala1979Val; p.Pro1898Arg; p.Leu2279Ile; p.Glu2055Ter	47	28
NF2	p.Thr180IlefsTer29; p.Glu206Ter; p.Ser288Ter; p.Ser267ArgfsTer27; c.1575-1G>T; p.Ile210Thr; p.Glu167Ter; c.114+2T>A; p.Phe22LeufsTer3; p.Glu182Ter; p.Arg57Ter; p.Tyr217_Val219delinsTer; p.Trp74Ter	13	13
NFRKB	p.Pro927Thr; p.Ala989Thr; p.Arg176Trp; c.891+1G>A; p.Pro764Leu; p.Pro527Leu; c.1460-7C>T; p.Thr1134Asn; p.Ala1033Val; p.Asp737Asn; p.His821Gln	11	9
NOD2	p.Ala781Val; p.Leu701Phe; p.Asp824Asn; p.Arg391His; p.Val259Ile; p.Arg543His; p.Pro80Ser; p.Ala819Thr; p.Ala266Thr; p.Cys354Tyr; p.Pro528GlnfsTer235	12	11
PIK3CA	p.Arg524Lys; p.Asn372Lys; p.Leu244Ter; p.Ala1066Thr; p.Glu218Ter; p.Glu418Lys; p.Pro421Ser; p.Thr470Ile	12	10
PIK3CB	p.Glu225Lys; p.Gly28Asp; p.Ser521Asn; p.Arg669Ser; p.Gly971Ter; c.2797-4C>T	6	5
PSMD13	p.Arg156His	1	1
RAPGEF6	c.4489+5G>T; p.Ser1440Ile; p.Pro633Ser; p.Met993Ile; p.Arg902Met; p.Asn1565Ser; p.Ala1113Thr; p.Lys926AsnfsTer3; p.Thr285Ile	9	5
RDX	p.Lys448Glu; p.Asn112Lys; p.Pro119Thr; c.959+1G>A; p.Lys3AsnfsTer5; p.Gly286Val	42	29
SETDB1	p.Gly1010Glu; p.Gly891Ser; p.Glu1011Lys; p.His77GlnfsTer3; p.Pro713Gln; p.Ala560Thr; p.Pro950Ser; c.3130-5T>G; p.Ala23Val; p.Ala833Val; p.Thr66Lys	13	10
TAOK1	p.Cys215Ser; p.Ser717Cys; p.Glu249Lys; c.750-1G>T	4	3
TP53	p.Thr55AsnfsTer71; p.Ile195Thr; p.Ala161Thr; p.Val173Ala; p.Arg249Ser; p.Val272Leu; p.Cys242Tyr; p.Met160SerfsTer6; p.Ser241_Gly245del; p.Arg213HisfsTer34; p.Pro359Thr	11	8
TXNRD1	p.Arg501Ile; p.Ala383Val	2	2
XRCC6	p.Glu333Ter; p.Gly127Glu; p.Pro532Gln; p.Thr541Ile; p.Pro175Thr	5	5

**Table 2:** Comparison of genes mutation frequencies in epithelioid and biphasic mesotheliomas. WT: Wild Type.

	Epithelioid (*n* = 30)	Biphasic (*n* = 39)	Total (*n* = 69)	*p* Value
**ACTB**				1.000
WT	30 (100.0%)	38 (97.4%)	68 (98.6%)	
Mutated	0 (0.0%)	1 (2.6%)	1 (1.4%)	
**ACTG1**				0.002
WT	21 (70.0%)	38 (97.4%)	59 (85.5%)	
Mutated	9 (30.0%)	1 (2.6%)	10 (14.5%)	
**ACTG2**				0.252
WT	30 (100.0%)	36 (92.3%)	66 (95.7%)	
Mutated	0 (0.0%)	3 (7.7%)	3 (4.3%)	
**ACTR1A**				0.501
WT	30 (100.0%)	37 (94.9%)	67 (97.1%)	
Mutated	0 (0.0%)	2 (5.1%)	2 (2.9%)	
**BAP1**				0.238
WT	21 (70.0%)	33 (84.6%)	54 (78.3%)	
Mutated	9 (30.0%)	6 (15.4%)	15 (21.7%)	
**CDH8**				0.064
WT	30 (100.0%)	34 (87.2%)	64 (92.8%)	
Mutated	0 (0.0%)	5 (12.8%)	5 (7.2%)	
**COL3A1**				1.000
WT	27 (90.0%)	36 (92.3%)	63 (91.3%)	
Mutated	3 (10.0%)	3 (7.7%)	6 (8.7%)	
**COL5A2**				0.690
WT	28 (93.3%)	34 (87.2%)	62 (89.9%)	
Mutated	2 (6.7%)	5 (12.8%)	7 (10.1%)	
**CUL1**				0.252
WT	30 (100.0%)	36 (92.3%)	66 (95.7%)	
Mutated	0 (0.0%)	3 (7.7%)	3 (4.3%)	
**GOT1**				1.000
WT	29 (96.7%)	37 (94.9%)	66 (95.7%)	
Mutated	1 (3.3%)	2 (5.1%)	3 (4.3%)	
**KDR**				0.627
WT	29 (96.7%)	36 (92.3%)	65 (94.2%)	
Mutated	1 (3.3%)	3 (7.7%)	4 (5.8%)	
**KIT**				0.690
WT	28 (93.3%)	34 (87.2%)	62 (89.9%)	
Mutated	2 (6.7%)	5 (12.8%)	7 (10.1%)	
**MXRA5**				0.050
WT	22 (73.3%)	19 (48.7%)	41 (59.4%)	
Mutated	8 (26.7%)	20 (51.3%)	28 (40.6%)	
**NF2**				0.128
WT	27 (90.0%)	29 (74.4%)	56 (81.2%)	
Mutated	3 (10.0%)	10 (25.6%)	13 (18.8%)	
**NFRKB**				0.722
WT	27 (90.0%)	33 (84.6%)	60 (87.0%)	
Mutated	3 (10.0%)	6 (15.4%)	9 (13.0%)	
**NOD2**				0.018
WT	29 (96.7%)	29 (74.4%)	58 (84.1%)	
Mutated	1 (3.3%)	10 (25.6%)	11 (15.9%)	
**PIK3CA**				0.090
WT	23 (76.7%)	36 (92.3%)	59 (85.5%)	
Mutated	7 (23.3%)	3 (7.7%)	10 (14.5%)	
**PIK3CB**				1.000
WT	28 (93.3%)	36 (92.3%)	64 (92.8%)	
Mutated	2 (6.7%)	3 (7.7%)	5 (7.2%)	
**PSMD13**				1.000
WT	30 (100.0%)	38 (97.4%)	68 (98.6%)	
Mutated	0 (0.0%)	1 (2.6%)	1 (1.4%)	
**RAPGEF6**				0.379
WT	29 (96.7%)	35 (89.7%)	64 (92.8%)	
Mutated	1 (3.3%)	4 (10.3%)	5 (7.2%)	
**RDX**				0.624
WT	16 (53.3%)	24 (61.5%)	40 (58.0%)	
Mutated	14 (46.7%)	15 (38.5%)	29 (42.0%)	
**SETDB1**				1.000
WT	26 (86.7%)	33 (84.6%)	59 (85.5%)	
Mutated	4 (13.3%)	6 (15.4%)	10 (14.5%)	
**TAOK1**				0.077
WT	27 (90.0%)	39 (100.0%)	66 (95.7%)	
Mutated	3 (10.0%)	0 (0.0%)	3 (4.3%)	
**TP53**				0.720
WT	26 (86.7%)	35 (89.7%)	61 (88.4%)	
Mutated	4 (13.3%)	4 (10.3%)	8 (11.6%)	
**TXNRD1**				0.501
WT	30 (100.0%)	37 (94.9%)	67 (97.1%)	
Mutated	0 (0.0%)	2 (5.1%)	2 (2.9%)	
**XRCC6**				0.064
WT	30 (100.0%)	34 (87.2%)	64 (92.8%)	
Mutated	0 (0.0%)	5 (12.8%)	5 (7.2%)	
**mutated genes/patient**				0.331
Mean (SD)	3.282 (2.625)	2.567 (1.736)	2.971 (2.294)	

**Table 3:** Univariate and multivariate survival analysis of biphasic mesotheliomas.

	Univariate Analysis			Multivariate Analysis		
	HR	C.I. 95%	*p* Value	HR	C.I. 95%	*p* Value
**MXRA5**	0.95	0.47–1.91	0.884	1.76	0.63–4.92	0.279
**NOD2**	0.93	0.42–2.05	0.852	0.21	0.06–0.79	**0.021**
**CDH8**	2.2	0.74–6.53	0.156	2.15	0.26–17.8	0.478
**XRCC6**	1.2	0.51–2.30	**0.022**	8.16	1.16–57.2	**0.035**
**Surgery**	0.43	0.20–0.95	**0.036**	0.85	0.28–2.58	0.775
**Performance Status**	3.89	1.44–10.49	**0.007**	4.45	1.04–19.0	**0.043**
**TNM stage 1 vs.** **TNM stage 2-3-4**	1.32	0.67–2.62	0.423	1.27	0.52–3.13	0.602
**FEV1% (30–80 vs. >80%)**	0.59	0.25–1.37	0.223	0.35	0.11–1.13	0.078

## Supplementary Materials

The following are available online at https://www.mdpi.com/2072-6694/12/9/2454/s1. Table S1: Clinical features of 69 epithelioid and biphasic mesothelioma patients, Table S2: Comparison of genes mutation frequencies in mesothelioma tumors at different pathological stage, Table S3: Clinical features of 11 sarcomatoid mesothelioma patients, Table S4: List of mutations detected in sarcomatoid MPM, Table S5: Genes mutation frequencies in sarcomatoid mesotheliomas, Table S6: List of genes included in next generation sequencing panel. Figure S1: Analysis of the impact of co-occurring mutations on tumor stage. Figure S2: Mutational status of a validation cohort of 16 biphasic mesotheliomas.
