# Supplementary material for: Deep Sequencing Analysis Identified a Specific Subset of Mutations Distinctive of Biphasic Malignant Pleural Mesothelioma
Source: Cancers (Basel). 2020 Aug 29;12(9):2454. doi: 10.3390/cancers12092454 (PMC7563974; doi:10.3390/cancers12092454)
Supplement: Supplementary file 1 [file cancers-12-02454-s001.zip › Supplementary files/Figure S1.pdf]

FIGURE S1

A

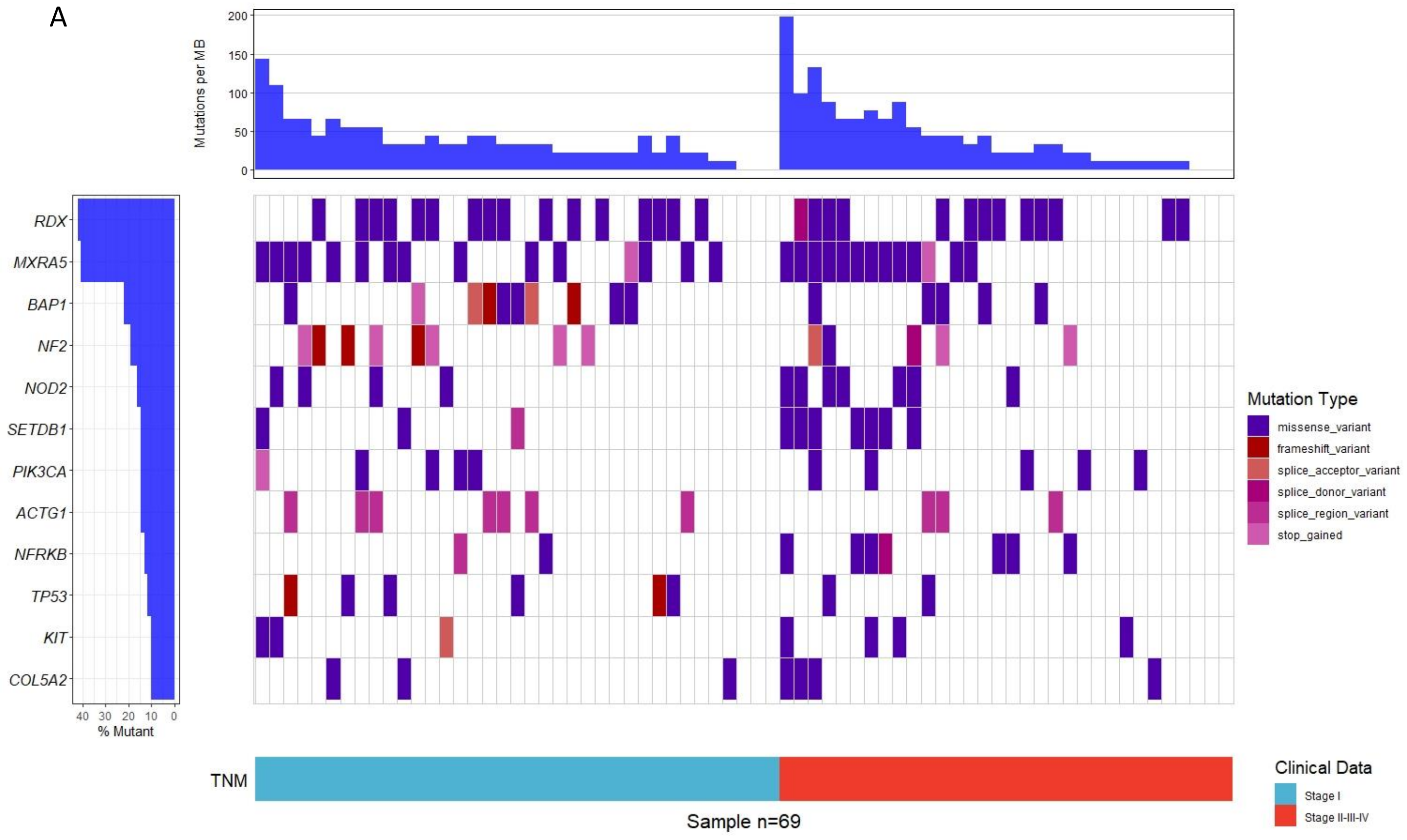

B

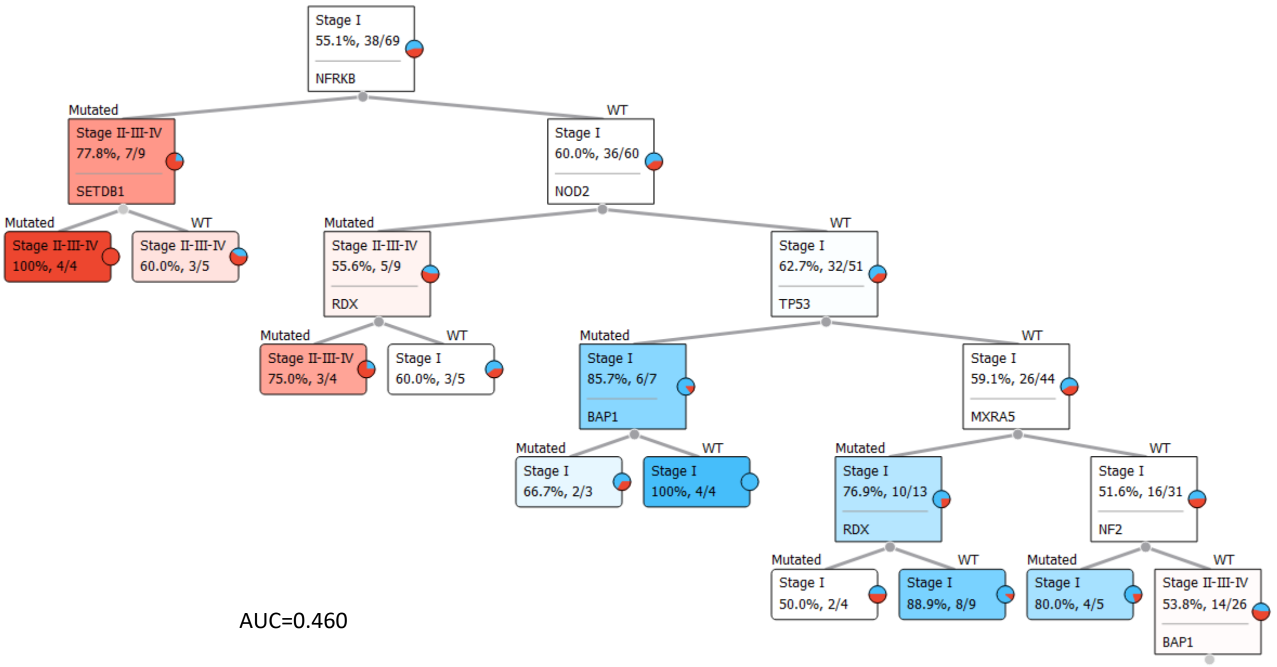

FIGURE S1. Analysis of the impact of co-occurring mutations on tumor stage.

A. Waterfall plot representing the gene mutations co-occurred in each patient (columns). Patients were subdivided by TNM stage as indicated by lower horizontal bar.
B. Decision tree showing the role of gene mutations co-occurrence in the classification of MPM by stage. AUC of the method was calculated by a 10-fold cross validation.
