# Supplementary material for: Deep Sequencing Analysis Identified a Specific Subset of Mutations Distinctive of Biphasic Malignant Pleural Mesothelioma
Source: Cancers (Basel). 2020 Aug 29;12(9):2454. doi: 10.3390/cancers12092454 (PMC7563974; doi:10.3390/cancers12092454)
Supplement: Supplementary file 1 [file cancers-12-02454-s001.zip › Supplementary files/Figure S2.pdf]

FIGURE S2

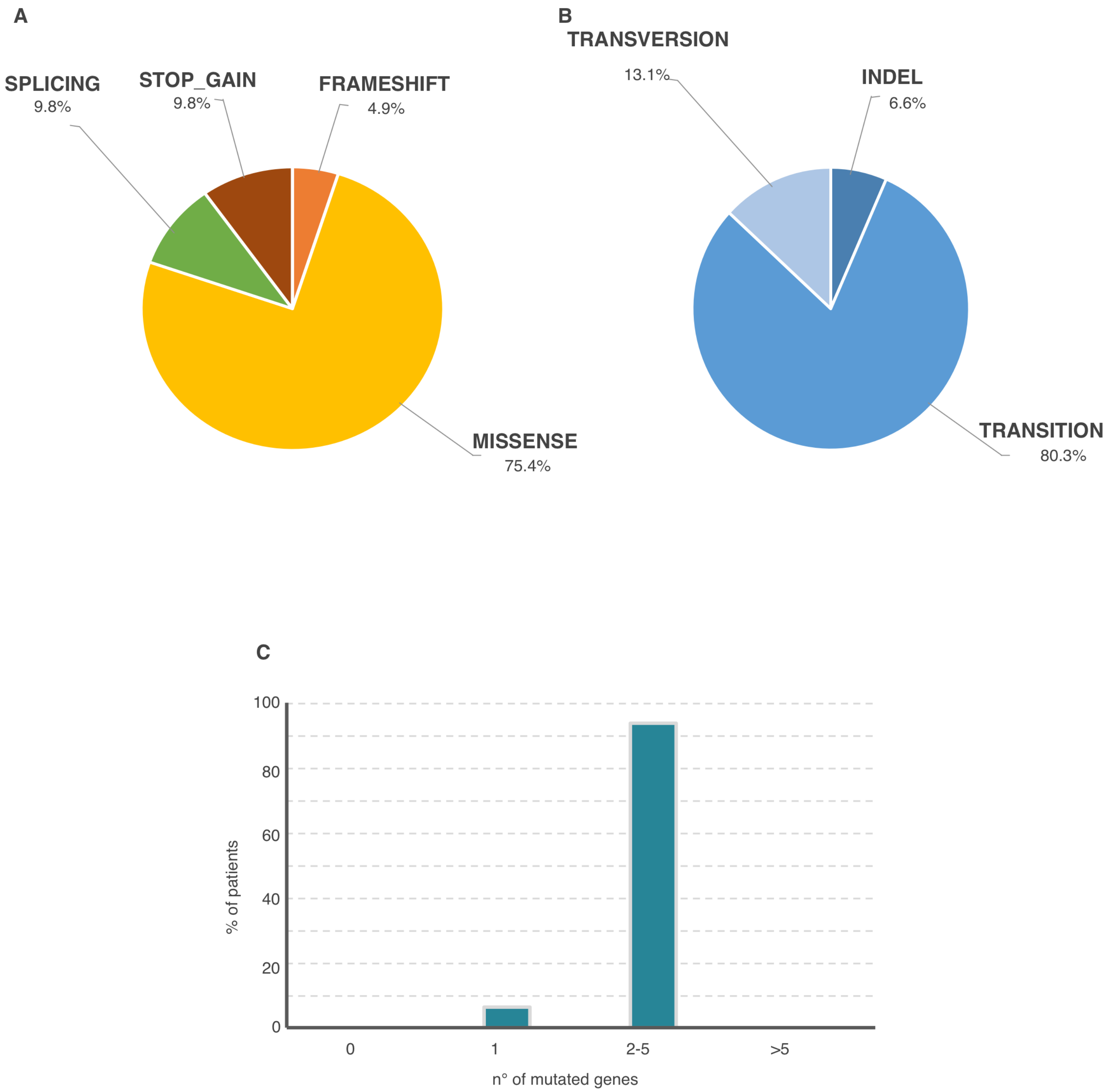

FIGURE S2. Mutational status of a validation cohort of 16 biphasic mesotheliomas.

A. Distribution of mutation types.

B. Distribution of mutation coding effects.

C. Distribution of the number of mutated genes per patient.
