# Supplementary material for: Deep Sequencing Analysis Identified a Specific Subset of Mutations Distinctive of Biphasic Malignant Pleural Mesothelioma
Source: Cancers (Basel). 2020 Aug 29;12(9):2454. doi: 10.3390/cancers12092454 (PMC7563974; doi:10.3390/cancers12092454)
Supplement: Supplementary file 1 [file cancers-12-02454-s001.zip › Supplementary files/Table S1.docx]

|  | **Overall (N=69)** |
| --- | --- |
| **Age** |  |
| Mean (SD) | 68.1 (9.8) |
| **Gender** |  |
| F | 19 (27.5%) |
| M | 50 (72.5%) |
| **Histology** |  |
| Biphasic | 39 (56.5%) |
| Epithelioid | 30 (43.5%) |
| **Asbestos exposure^§^** |  |
| Direct exposure | 40 (77.0%) |
| Indirect exposure | 6 (11.5%) |
| No exposure | 6 (11.5%) |
| **Smoking Habit^§^** |  |
| ex smoker | 15 (25.0%) |
| No | 20 (33.3%) |
| Smoker | 25 (41.7%) |
| **Comorbidities^§^** |  |
| No | 17 (30.4%) |
| Yes | 39 (69.6%) |
| **Side** |  |
| Both | 1 (1.4%) |
| left | 33 (47.8%) |
| right | 35 (50.7%) |
| **Surgery** |  |
| No | 43 (62.3%) |
| Yes | 26 (37.7%) |
| **TNM** |  |
| I  II  III  IV | 37 (53.6%)  2 (2.9%)  28 (40.6%)  2 (2.9%) |
| **Neoadjuvant therapy** |  |
| No | 60 (87.0%) |
| Yes | 9 (13.0%) |

§Missing data

**Table S1.** Clinical features of 69 epithelioid and biphasic mesothelioma patients.
