# Supplementary material for: Deep Sequencing Analysis Identified a Specific Subset of Mutations Distinctive of Biphasic Malignant Pleural Mesothelioma
Source: Cancers (Basel). 2020 Aug 29;12(9):2454. doi: 10.3390/cancers12092454 (PMC7563974; doi:10.3390/cancers12092454)
Supplement: Supplementary file 1 [file cancers-12-02454-s001.zip › Supplementary files/Table S2.doc]

|  | **Stage I**  **(N=37)** | **Stage II-III-IV**  **(N=32)** | **Total**  **(N=69)** | **p value** |
| --- | --- | --- | --- | --- |
| **ACTB** |  |  |  | 0.464 |
| WT | 37 (100.0%) | 31 (96.9%) | 68 (98.6%) |  |
| Mutated | 0 (0.0%) | 1 (3.1%) | 1 (1.4%) |  |
| **ACTG1** |  |  |  | 0.320 |
| WT | 30 (81.1%) | 29 (90.6%) | 59 (85.5%) |  |
| Mutated | 7 (18.9%) | 3 (9.4%) | 10 (14.5%) |  |
| **ACTG2** |  |  |  | 0.593 |
| WT | 36 (97.3%) | 30 (93.8%) | 66 (95.7%) |  |
| Mutated | 1 (2.7%) | 2 (6.2%) | 3 (4.3%) |  |
| **ACTR1A** |  |  |  | 0.211 |
| WT | 37 (100.0%) | 30 (93.8%) | 67 (97.1%) |  |
| Mutated | 0 (0.0%) | 2 (6.2%) | 2 (2.9%) |  |
| **BAP1** |  |  |  | 0.381 |
| WT | 27 (73.0%) | 27 (84.4%) | 54 (78.3%) |  |
| Mutated | 10 (27.0%) | 5 (15.6%) | 15 (21.7%) |  |
| **CDH8** |  |  |  | 0.657 |
| WT | 35 (94.6%) | 29 (90.6%) | 64 (92.8%) |  |
| Mutated | 2 (5.4%) | 3 (9.4%) | 5 (7.2%) |  |
| **COL3A1** |  |  |  | 0.089 |
| WT | 36 (97.3%) | 27 (84.4%) | 63 (91.3%) |  |
| Mutated | 1 (2.7%) | 5 (15.6%) | 6 (8.7%) |  |
| **COL5A2** |  |  |  | 0.696 |
| WT | 34 (91.9%) | 28 (87.5%) | 62 (89.9%) |  |
| Mutated | 3 (8.1%) | 4 (12.5%) | 7 (10.1%) |  |
| **CUL1** |  |  |  | 1.000 |
| WT | 35 (94.6%) | 31 (96.9%) | 66 (95.7%) |  |
| Mutated | 2 (5.4%) | 1 (3.1%) | 3 (4.3%) |  |
| **GOT1** |  |  |  | 0.593 |
| WT | 36 (97.3%) | 30 (93.8%) | 66 (95.7%) |  |
| Mutated | 1 (2.7%) | 2 (6.2%) | 3 (4.3%) |  |
| **KDR** |  |  |  | 0.330 |
| WT | 36 (97.3%) | 29 (90.6%) | 65 (94.2%) |  |
| Mutated | 1 (2.7%) | 3 (9.4%) | 4 (5.8%) |  |
| **KIT** |  |  |  | 0.696 |
| WT | 34 (91.9%) | 28 (87.5%) | 62 (89.9%) |  |
| Mutated | 3 (8.1%) | 4 (12.5%) | 7 (10.1%) |  |
| **MXRA5** |  |  |  | 1.000 |
| WT | 22 (59.5%) | 19 (59.4%) | 41 (59.4%) |  |
| Mutated | 15 (40.5%) | 13 (40.6%) | 28 (40.6%) |  |
| **NF2** |  |  |  | 0.556 |
| WT | 29 (78.4%) | 27 (84.4%) | 56 (81.2%) |  |
| Mutated | 8 (21.6%) | 5 (15.6%) | 13 (18.8%) |  |
| **NFRKB** |  |  |  | 0.071 |
| WT | 35 (94.6%) | 25 (78.1%) | 60 (87.0%) |  |
| Mutated | 2 (5.4%) | 7 (21.9%) | 9 (13.0%) |  |
| **NOD2** |  |  |  | 0.324 |
| WT | 33 (89.2%) | 25 (78.1%) | 58 (84.1%) |  |
| Mutated | 4 (10.8%) | 7 (21.9%) | 11 (15.9%) |  |
| **PIK3CA** |  |  |  | 1.000 |
| WT | 32 (86.5%) | 27 (84.4%) | 59 (85.5%) |  |
| Mutated | 5 (13.5%) | 5 (15.6%) | 10 (14.5%) |  |
| **PIK3CB** |  |  |  | 0.363 |
| WT | 33 (89.2%) | 31 (96.9%) | 64 (92.8%) |  |
| Mutated | 4 (10.8%) | 1 (3.1%) | 5 (7.2%) |  |
| **PSMD13** |  |  |  | 0.464 |
| WT | 37 (100.0%) | 31 (96.9%) | 68 (98.6%) |  |
| Mutated | 0 (0.0%) | 1 (3.1%) | 1 (1.4%) |  |
| **RAPGEF6** |  |  |  | 0.057 |
| WT | 32 (86.5%) | 32 (100.0%) | 64 (92.8%) |  |
| Mutated | 5 (13.5%) | 0 (0.0%) | 5 (7.2%) |  |
| **RDX** |  |  |  | 1.000 |
| WT | 21 (56.8%) | 19 (59.4%) | 40 (58.0%) |  |
| Mutated | 16 (43.2%) | 13 (40.6%) | 29 (42.0%) |  |
| **SETDB1** |  |  |  | 0.170 |
| WT | 34 (91.9%) | 25 (78.1%) | 59 (85.5%) |  |
| Mutated | 3 (8.1%) | 7 (21.9%) | 10 (14.5%) |  |
| **TAOK1** |  |  |  | 0.593 |
| WT | 36 (97.3%) | 30 (93.8%) | 66 (95.7%) |  |
| Mutated | 1 (2.7%) | 2 (6.2%) | 3 (4.3%) |  |
| **TP53** |  |  |  | 0.270 |
| WT | 31 (83.8%) | 30 (93.8%) | 61 (88.4%) |  |
| Mutated | 6 (16.2%) | 2 (6.2%) | 8 (11.6%) |  |
| **TXNRD1** |  |  |  | 0.211 |
| WT | 37 (100.0%) | 30 (93.8%) | 67 (97.1%) |  |
| Mutated | 0 (0.0%) | 2 (6.2%) | 2 (2.9%) |  |
| **XRCC6** |  |  |  | 0.657 |
| WT | 35 (94.6%) | 29 (90.6%) | 64 (92.8%) |  |
| Mutated | 2 (5.4%) | 3 (9.4%) | 5 (7.2%) |  |
| **# mutated genes** |  |  |  |  |
| Mean (SD) | 2.757 (1.623) | 3.219 (2.893) | 2.971 (2.294) | 0.990 |

**Table S2.** Comparison of genes mutation frequencies in mesothelioma tumors at different pathological stage.
