# Supplementary material for: Deep Sequencing Analysis Identified a Specific Subset of Mutations Distinctive of Biphasic Malignant Pleural Mesothelioma
Source: Cancers (Basel). 2020 Aug 29;12(9):2454. doi: 10.3390/cancers12092454 (PMC7563974; doi:10.3390/cancers12092454)
Supplement: Supplementary file 1 [file cancers-12-02454-s001.zip › Supplementary files/Table S3.docx]

|  | **Overall (N=11)** | |
| --- | --- | --- |
| **Age** |  |  |
| Mean (SD) | 72.5(7.9) |  |
| **Gender** |  |  |
| F | 1 (9.1%) |  |
| M | 10 (90.9%) |  |
| **Asbestos exposure**§ |  |  |
| Direct exposure | 6 (100.0%) |  |
| Indirect exposure | 0 (0.0%) |  |
| no exposure | 0 (0.0%) |  |
| **Smoking Habit**§ |  |  |
| ex smoker | 0 (0.0%) |  |
| No | 2 (22.2%) |  |
| Smoker | 7 (77.8%) |  |
| **comorbidities**§ |  |  |
| No | 4 (57.1%) |  |
| Yes | 3 (42.9%) |  |
| **Side** |  |  |
| Both | 1 (9.1%) |  |
| left | 3 (27.3%) |  |
| right | 7 (63.6%) |  |
| **Surgery** |  |  |
| no | 11 (100.0%) |  |
| yes | 0 (0.0%) |  |
| **TNM** |  |  |
| I | 5 (45.5%) |  |
| II | 0 (0.0%) |  |
| III | 6 (54.5%) |  |
| IV | 0 (0.0%) |  |

§Missing data

**Table S3**. Clinical features of 11 sarcomatoid mesothelioma patients.
