# Supplementary material for: Deep Sequencing Analysis Identified a Specific Subset of Mutations Distinctive of Biphasic Malignant Pleural Mesothelioma
Source: Cancers (Basel). 2020 Aug 29;12(9):2454. doi: 10.3390/cancers12092454 (PMC7563974; doi:10.3390/cancers12092454)
Supplement: Supplementary file 1 [file cancers-12-02454-s001.zip › Supplementary files/Table S4.docx]

| **Gene** | **Mutations** | **Total number of mutation** | **Number of mutated patients** |
| --- | --- | --- | --- |
| **ACTG1** | c.984+7_984+9delCGA | 2 | 2 |
| **BAP1** | p.Thr69Lys | 1 | 1 |
| **KIT** | p.Thr245Met;p.Val530Ile | 2 | 2 |
| **MXRA5** | p.Leu1780Met; p.Glu893Lys; p.Arg2039Cys; p.Pro1561Ser | 4 | 2 |
| **NF2** | p.Ile487AsnfsTer8; p.Lys40SerfsTer83 | 2 | 2 |
| **NFRKB** | p.Pro848Leu | 1 | 1 |
| **NOD2** | p.Thr770Lys | 1 | 1 |
| **PIK3CA** | p.Val146Ile | 1 | 1 |
| **PIK3CB** | p.Asn331del | 1 | 1 |
| **RDX** | p.Pro119Thr; p.Asn112Lys; p.Tyr201Cys | 13 | 7 |
| **SETDB1** | p.Asp1132Tyr; p.Glu992Asp | 2 | 2 |
| **TAOK1** | p.Thr986Lys | 1 | 1 |
| **TP53** | p.Glu287Ter | 1 | 1 |

**Table S4**. List of mutations detected in sarcomatoid mesotheliomas.
