# Supplementary material for: Deep Sequencing Analysis Identified a Specific Subset of Mutations Distinctive of Biphasic Malignant Pleural Mesothelioma
Source: Cancers (Basel). 2020 Aug 29;12(9):2454. doi: 10.3390/cancers12092454 (PMC7563974; doi:10.3390/cancers12092454)
Supplement: Supplementary file 1 [file cancers-12-02454-s001.zip › Supplementary files/Table S5.docx]

|  | **Sarcomatoid (N=11)** |
| --- | --- |
| **ACTB** |  |
| WT | 11 (100.0%) |
| Mutated | 0 (0.0%) |
| **ACTG1** |  |
| WT | 9 (81.8%) |
| Mutated | 2 (18.2%) |
| **ACTG2** |  |
| WT | 11 (100.0%) |
| Mutated | 0 (0.0%) |
| **ACTR1A** |  |
| WT | 11 (100.0%) |
| Mutated | 0 (0.0%) |
| **BAP1** |  |
| WT | 10 (90.9%) |
| Mutated | 1 (9.1%) |
| **CDH8** |  |
| WT | 11 (100.0%) |
| Mutated | 0 (0.0%) |
| **COL3A1** |  |
| WT | 11 (100.0%) |
| Mutated | 0 (0.0%) |
| **COL5A2** |  |
| WT | 11 (100.0%) |
| Mutated | 0 (0.0%) |
| **CUL1** |  |
| WT | 11 (100.0%) |
| Mutated | 0 (0.0%) |
| **GOT1** |  |
| WT | 11 (100.0%) |
| Mutated | 0 (0.0%) |
| **KDR** |  |
| WT | 11 (100.0%) |
| Mutated | 0 (0.0%) |
| **KIT** |  |
| WT | 9 (81.8%) |
| Mutated | 2 (18.2%) |
| **MXRA5** |  |
| WT | 9 (81.8%) |
| Mutated | 2 (18.2%) |
| **NF2** |  |
| WT | 9 (81.8%) |
| Mutated | 2 (18.2%) |
| **NFRKB** |  |
| WT | 10 (90.9%) |
| Mutated | 1 (9.1%) |
| **NOD2** |  |
| WT | 10 (90.9%) |
| Mutated | 1 (9.1%) |
| **PIK3CA** |  |
| WT | 10 (90.9%) |
| Mutated | 1 (9.1%) |
| **PIK3CB** |  |
| WT | 10 (90.9%) |
| Mutated | 1 (9.1%) |
| **PSMD13** |  |
| WT | 11 (100.0%) |
| Mutated | 0 (0.0%) |
| **RAPGEF6** |  |
| WT | 11 (100.0%) |
| Mutated | 0 (0.0%) |
| **RDX** |  |
| WT | 4 (36.4%) |
| Mutated | 7 (63.6%) |
| **SETDB1** |  |
| WT | 9 (81.8%) |
| Mutated | 2 (18.2%) |
| **TAOK1** |  |
| WT | 10 (90.9%) |
| Mutated | 1 (9.1%) |
| **TP53** |  |
| WT | 10 (90.9%) |
| Mutated | 1 (9.1%) |
| **TXNRD1** |  |
| WT | 11 (100.0%) |
| Mutated | 0 (0.0%) |
| **XRCC6** |  |
| WT | 11 (100.0%) |
| Mutated | 0 (0.0%) |
| **# mutated genes** |  |
| Mean (SD) | 2.182 (1.328) |

**Table S5.** Genes mutation frequencies in sarcomatoid mesotheliomas
