# Supplementary material for: Deep Sequencing Analysis Identified a Specific Subset of Mutations Distinctive of Biphasic Malignant Pleural Mesothelioma
Source: Cancers (Basel). 2020 Aug 29;12(9):2454. doi: 10.3390/cancers12092454 (PMC7563974; doi:10.3390/cancers12092454)
Supplement: Supplementary file 1 [file cancers-12-02454-s001.zip › Supplementary files/Table S6.docx]

| **ACTB** | *Actin Beta* |
| --- | --- |
| **ACTG1** | *Actin Gamma 1* |
| **ACTG2** | *Actin Gamma 2* |
| **ACTR1A** | *ARP1 Actin Related Protein 1 Homolog A* |
| **BAP1** | *BRCA1 Associated Protein 1* |
| **CDH8** | *Cadherin 8* |
| **CDK4** | *Cyclin Dependent Kinase 4* |
| **CDKN2A** | *Cyclin Dependent Kinase Inhibitor 2A* |
| **CDKN2B** | *Cyclin Dependent Kinase Inhibitor 2B* |
| **COL3A1** | *Collagen Type III Alpha 1 Chain* |
| **COL5A2** | *COL5A2* |
| **CUL1** | *Cullin 1* |
| **DHFR** | *Dihydrofolate Reductase* |
| **GOT1** | *Glutamic-Oxaloacetic Transaminase 1* |
| **KDR** | *Kinase Insert Domain Receptor* |
| **KIT** | *KIT Proto-Oncogene Receptor Tyrosine Kinase* |
| **MXRA5** | *Matrix Remodeling Associated 5* |
| **NF2** | *Neurofibromin 2* |
| **NFRKB** | *Nuclear Factor Related To KappaB Binding Protein* |
| **NKX6-2** | *NK6 Homeobox 2* |
| **NOD2** | *Nucleotide Binding Oligomerization Domain Containing 2* |
| **PCBD2** | *Pterin-4 Alpha-Carbinolamine Dehydratase 2* |
| **PDZK1IP1** | *PDZK1 Interacting Protein 1* |
| **PIK3CA** | *Phosphatidylinositol-4,5-Bisphosphate 3-Kinase Catalytic Subunit Alpha* |
| **PIK3CB** | *Phosphatidylinositol-4,5-Bisphosphate 3-Kinase Catalytic Subunit Beta* |
| **PSMD13** | *Proteasome 26S Subunit, Non-ATPase 13* |
| **RAPGEF6** | *Rap Guanine Nucleotide Exchange Factor 6* |
| **RDX** | *Radixin* |
| **SETDB1** | *SET Domain Bifurcated 1* |
| **TAOK1** | *TAO Kinase 1* |
| **TP53** | *Tumor Protein P53* |
| **TXNRD1** | *Thioredoxin Reductase* |
| **UQCRC1** | *Ubiquinol-Cytochrome C Reductase Core Protein 1* |
| **XRCC6** | *X-Ray Repair Cross Complementing 6* |

**Table S6.** List of genes included in next generation sequencing panel
